# The Microbiome, Epigenome, and Diet in Adults with Obesity during Behavioral Weight Loss

**DOI:** 10.3390/nu15163588

**Published:** 2023-08-16

**Authors:** Emily B. Hill, Iain R. Konigsberg, Diana Ir, Daniel N. Frank, Purevsuren Jambal, Elizabeth M. Litkowski, Ethan M. Lange, Leslie A. Lange, Danielle M. Ostendorf, Jared J. Scorsone, Liza Wayland, Kristen Bing, Paul S. MacLean, Edward L. Melanson, Daniel H. Bessesen, Victoria A. Catenacci, Maggie A. Stanislawski, Sarah J. Borengasser

**Affiliations:** 1 Section of Nutrition, Department of Pediatrics, University of Colorado Anschutz Medical Campus, Aurora, CO 80045, USA; emily.b.hill@cuanschutz.edu (E.B.H.);; 2 Department of Biomedical Informatics, University of Colorado Anschutz Medical Campus, Aurora, CO 80045, USA; iain.konigsberg@cuanschutz.edu (I.R.K.);; 3 Division of Infectious Diseases, Department of Medicine, University of Colorado Anschutz Medical Campus, Aurora, CO 80045, USA; 4 Department of Epidemiology, University of Colorado School of Public Health, Aurora, CO 80045, USA; 5 Eastern Colorado Veterans Affairs Geriatric Research, Education, and Clinical Center, Aurora, CO 80045, USA; 6 Department of Biostatistics and Informatics, University of Colorado School of Public Health, Aurora, CO 80045, USA; 7 Division of Endocrinology, Metabolism, and Diabetes, Department of Medicine, University of Colorado Anschutz Medical Campus, Aurora, CO 80045, USA; 8 Anschutz Health and Wellness Center, Department of Medicine, University of Colorado Anschutz Medical Campus, Aurora, CO 80045, USA; 9 Division of Geriatric Medicine, Department of Medicine, University of Colorado Anschutz Medical Campus, Aurora, CO 80045, USA

**Keywords:** DNA methylation, epigenetics, gut microbiome, diet, lifestyle, obesity

## Abstract

Obesity has been linked to the gut microbiome, epigenome, and diet, yet these factors have not been studied together during obesity treatment. Our objective was to evaluate associations among gut microbiota (MB), DNA methylation (DNAme), and diet prior to and during a behavioral weight loss intervention. Adults (*n* = 47, age 40.9 ± 9.7 years, body mass index (BMI) 33.5 ± 4.5 kg/m^2^, 77% female) with data collected at baseline (BL) and 3 months (3 m) were included. Fecal MB was assessed via 16S sequencing and whole blood DNAme via the Infinium EPIC array. Food group and nutrient intakes and Healthy Eating Index (HEI) scores were calculated from 7-day diet records. Linear models were used to test for the effect of taxa relative abundance on DNAme and diet cross-sectionally at each time point, adjusting for confounders and a false discovery rate of 5%. Mean weight loss was 6.2 ± 3.9% at 3 m. At BL, one MB taxon, *Ruminiclostridium*, was associated with DNAme of the genes *COL20A1* (r = 0.651, *p* = 0.029), *COL18A1* (r = 0.578, *p* = 0.044), and *NT5E* (r = 0.365, *p* = 0.043). At 3 m, there were 14 unique MB:DNAme associations, such as *Akkermansia* with DNAme of *GUSB* (r = −0.585, *p* = 0.003), *CRYL1* (r = −0.419, *p* = 0.007), *C9* (r = −0.439, *p* = 0.019), and *GMDS* (r = −0.559, *p* = 0.046). Among taxa associated with DNAme, no significant relationships were seen with dietary intakes of relevant nutrients, food groups, or HEI scores. Our findings indicate that microbes linked to mucin degradation, short-chain fatty acid production, and body weight are associated with DNAme of phenotypically relevant genes. These relationships offer an initial understanding of the possible routes by which alterations in gut MB may influence metabolism during weight loss.

## 1. Introduction

Modifiable factors, such as diet and physical activity patterns, influence both prevention and treatment of obesity and its sequalae. However, underlying mechanisms by which lifestyle affects weight status are complex. Potential etiologic pathways in obesity development may include changes in gene expression due to environmentally induced epigenetic modifications that lead to reprogramming of endocrine or metabolic regulator circuits [1,2]. DNA methylation (DNAme) profiles also change in response to lifestyle interventions, suggesting that alterations in nutrition or exercise may favorably modulate gene activity to improve phenotypes during obesity treatment [3]. In addition, the gut microbiota (MB) is involved in several physiological functions that maintain metabolic homeostasis, including mechanisms involving energy balance, inflammation, and appetite regulation [4]. Compositional and functional changes in the MB through environmental or lifestyle exposures, such as diet may, therefore, impact the development of obesity and cardiometabolic disease [5,6,7,8,9]. There is evidence to suggest that the gut MB may contribute towards weight loss, indicating that the gut MB may serve as useful therapeutic target for obesity management [10,11].

While the gut MB and epigenome may independently act to influence weight and other indicators of health in response to lifestyle, little is known about the interplay between them [12]. It is possible that microbes may themselves contribute to weight regulation through direct interaction with host cells to influence metabolism or through microbial signaling that influences other metabolic factors [13]. It has also been proposed that circulating metabolites may not only lead to beneficial metabolic effects but could also provide a link between the gut MB and epigenetic changes [14,15,16,17]. However, this relationship is poorly understood, with few primary research articles investigating the relationship between these two measures and limited mechanistic understanding of microbiota-host epigenome interactions [15,16].

Though previous literature suggests both the gut MB and epigenome are responsive to changes in lifestyle, these factors have not been considered together in the context of a behavioral weight loss intervention. Thus, the primary objective of the present study was to evaluate associations among the gut MB and DNAme prior to and during a behavioral weight loss intervention, with a secondary objective to evaluate the relationship between these measures, as well as a targeted plasma metabolomics panel and diet. We hypothesized that DNAme of genes within metabolically relevant pathways would be associated with MB composition and that these taxa would be responsive to changes in dietary intakes within 3 months of initiating a behavioral weight loss intervention. Evaluation of these relationships will aid in hypothesis generation for follow-up studies investigating how the gut MB influences metabolism during weight loss.

## 2. Materials and Methods

### 2.1. Participants and Study Design

**Figure 1:**
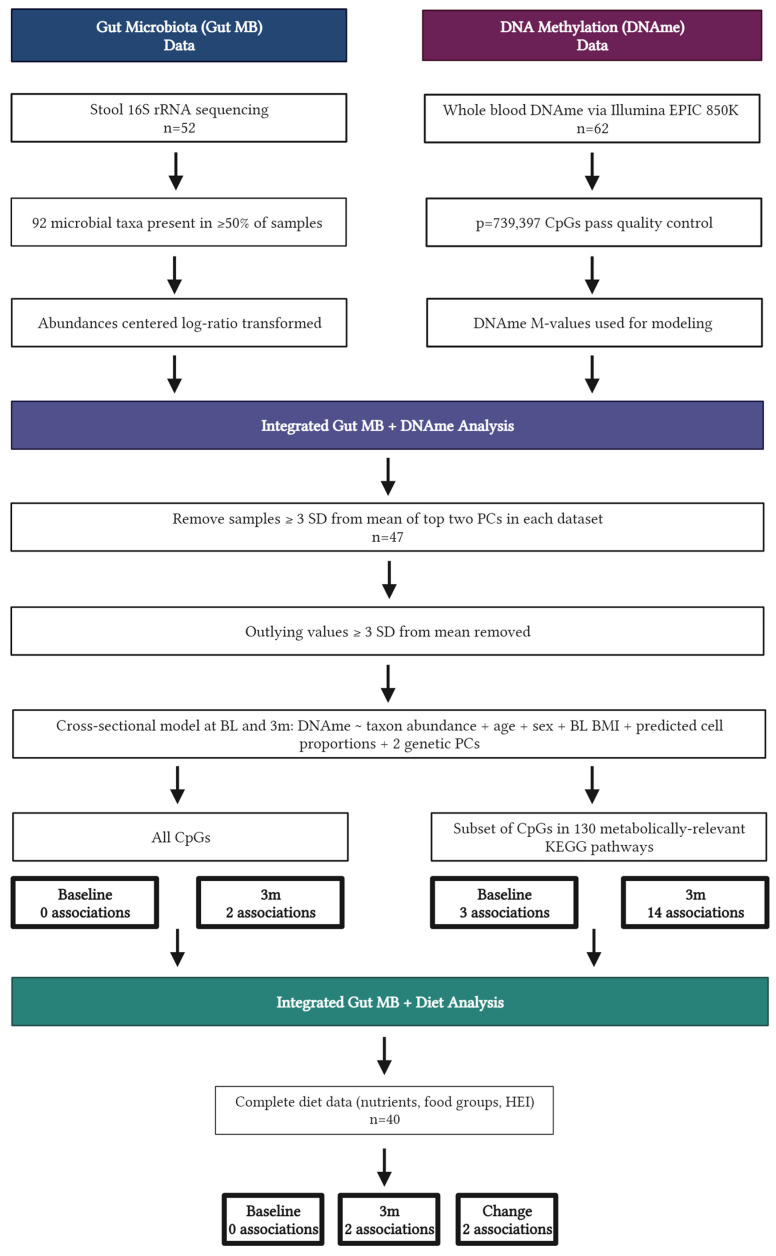
Flow diagram displaying data analysis for *n* = 47 individuals, created with BioRender.com.

Participants in this ancillary study were healthy individuals with overweight or obesity (age 18–55 years, BMI 27–45 kg/m^2^) enrolled in a randomized controlled trial (NCT03411356) comparing weight loss generated by daily caloric restriction (DCR) or intermittent fasting (IMF) during a one-year behavioral weight loss intervention. All participants were provided comprehensive behavioral support, encouraged to reduce calorie intake by ~34% per week from baseline estimated energy requirements, and increase physical activity to 300 min of moderate-to-vigorous activity per week. Stool samples, whole blood, clinical, and dietary intake data were collected at in-person assessments, and individuals from both arms of the first two of five cohorts of enrolled participants (starting April 2018 through February 2019) who had complete biospecimen collection at baseline and an early intervention time point of 3 months and agreed to optional ancillary studies were included in the present study. All study procedures were reviewed and approved by the Colorado Multiple Institutional Review Board, and individuals provided written, informed consent. Additional details regarding study procedures have been previously published [11,18,19]. Forty-seven participants provided viable stool and blood samples after data quality control (Figure 1).

### 2.2. Data Collection

#### 2.2.1. Clinical Assessments and Anthropometrics

Individuals attended assessment visits at baseline and 3 months. Prior to visits, participants were asked to fast for at least 12 h. Anthropometry measures, including weight and waist circumference, clinical assessments, including systolic blood pressure and diastolic blood pressure, blood collection and processing, and cardiometabolic assays were completed according to standard protocols by trained research personnel at the University of Colorado Anschutz Health and Wellness Center (AHWC) and the University of Colorado Clinical and Translational Research Center (CTRC). Weight was obtained using a digital scale accurate to 0.1 kg, and height measured with a fixed stadiometer to the nearest 1 mm at baseline only. Waist circumference was measured in duplicate to the nearest 1 mm using a tape measure at the top of the iliac crest, and the average was recorded. Blood pressure was measured using a manual sphygmomanometer using the average of two seated values taken after five minutes of rest. Participants self-collected stool at home using the Alpco EasySampler^®^ Stool Collection Kit (ALPCO Diagnostics, Salem, NH, USA) and provided samples to study staff at clinic visits. Briefly, ~1–2 g of stool were collected, sterilely transferred to provided collection tubes, and stored at −20 °C in home freezers prior to transfer to the clinic on ice packs within one week of collection at baseline and 3 months. Whole blood and stool samples were stored at −80 °C until subsequent analysis for DNAme and gut MB sequencing, respectively.

#### 2.2.2. DNA Methylation and Data Pre-Processing

Genomic DNA was isolated from whole blood samples using the commercially available PureLink Genomic DNA Kit (Invitrogen, Carlsbad, CA, USA). Quality was assessed via a NanoDrop 2000/2000c spectrophotometer (ThermoFisher Scientific, Grand Island, NY, USA) and concentrations were measured by a Qubit Fluorometer 2.0 (Invitrogen, Carlsbad, CA, USA). DNAme was assessed via the Illumina Infinium Human Methylation EPIC BeadChip Array (EPIC 850K) at the University of Colorado Anschutz Medical Campus Genomics and Microarray Core. DNA samples were bisulfite converted using the EZ DNA Methylation Kit (Zymo Research, Irvine, CA, USA) and used as inputs for the EPIC 850K array. Positive and negative controls from each conversion assay were included, and data were visualized using GenomeStudio Software (Illumina, Inc., San Diego, CA, USA). 

Array data were pre-processed and normalized using the SeSAMe R package [20]. After removing cross-hybridizing probes, probes overlying SNPs with minor allele frequency >1% in the general population [21], and probes that failed across multiple samples with an average detection *p*-value > 0.05, a total of 739,397 cytosine–phosphate–guanine dinucleotide (CpG) probes remained for analysis. Cell type frequencies (percentage of CD8 T cells, CD4 T cells, NK cells, B cells, monocytes, and neutrophils) were estimated using estimateCellCounts with the IlluminaHumanMethylationEPIC reference panel in the minfi R package [22]. 

All CpGs from the 850K array that passed quality control were included in subsequent analyses. In addition, a sensitivity analysis using only CpGs within genes mapped to 130 Kyoto Encyclopedia of Genes and Genomes (KEGG) pathways involved in metabolism (e.g., lipid and carbohydrate metabolism), organismal systems (e.g., digestive, endocrine, and immune systems), and human diseases (e.g., metabolic disease, cardiovascular disease) and, therefore, deemed highly relevant to overweight and obesity, was also completed (Table S1). This resulted in 82,889 CpG probes included in the sensitivity analyses. Array fluorescent intensities were transformed into M-values for statistical analysis [23]. 

#### 2.2.3. Microbiome Sequencing and Pre-Processing

Stool samples were thawed from −80 °C on ice and homogenized using the Roche MagNA Lyser (Roche, Inc., Basel, Switzerland) and DNA was extracted using the QiaAmp PowerFecal DNA kit (Qiagen, Hilden, Germany). To assess bacterial composition, amplification and sequence analysis of 16S rRNA genes was conducted as previously described [24,25]. Amplicons were generated using primers targeting base pairs within the V3V4 variable region of the 16S rRNA gene. PCR products were normalized using a SequalPrep™ kit (Invitrogen, Carlsbad, CA, USA), pooled, lyophilized, and then purified and concentrated using the DNA Clean and Concentrator Kit (Zymo, Irvine, CA, USA). The Qubit Fluorometer 2.0 was used to quantify pooled amplicons prior to sequencing. In brief, the amplicon pool was diluted to 4 nM and denatured using 0.2 N NaOH at room temperature. Denatured DNA was diluted to 15 pM and spiked with 25% Illumina PhiX control DNA prior to performing paired-end sequencing using the MiSeq platform (version 2.4) with a 600-cycle version 3 reagent kit.

All reads were quality filtered and trimmed to a uniform length using the average position of the first low-quality base pair among all samples using Qiime2 software version 2019.10 [26]. The data were de-noised using DADA2 under default parameters [27], and quality-filtered sequences were inserted into the SILVA 12.8 taxonomic database [28] using the SATé-enabled phylogenetic placement (SEPP) technique [29]. Analyses were standardized at 3407 sequences per sample. Microbe abundances were center log-ratio transformed, and after quality control to remove taxa present in <50% of samples, 92 genus-level taxa were retained for analysis.

#### 2.2.4. Dietary Intake Assessment and Data Pre-Processing

Participants completed 7-day diet records at baseline and 3 months. Individuals were asked to record all foods and beverages consumed each day, including detail on brand names, preparation, cooking methods, and portion sizes in household measurements. Diet records were analyzed by trained registered dietitian nutritionists (RDNs) at the Colorado Clinical and Translational Sciences Institute (CCTSI) Nutrition Core using the Nutrition Data System for Research (NDSR) nutrient analysis software versions 2018 and 2019 to align with year of data collection, developed by the Nutrition Coordinating Center (NCC) at the University of Minnesota, Minneapolis, MD, USA. 

Daily intake of energy, macronutrients, fiber, micronutrients, and NCC food group serving counts were exported from NDSR to calculate mean intakes over seven days at each time point. Individual food subgroups (e.g., citrus juice, fruit excluding citrus fruit) were further collapsed into food groups (e.g., fruit) based upon NCC food group serving count subgroup IDs. Nutrient and food group data were used to calculate Healthy Eating Index (HEI) total and component scores using all seven days of intake for each time point using published methods for the simple HEI scoring algorithm and publicly available SAS code [30]. Briefly, the HEI measures adherence to the US Dietary Guidelines for Americans, and total scores represent energy adjusted intakes of key food groups for adequacy (9 components) and moderation (4 components). Total scores range from 0–100, with higher scores reflective of greater adherence to dietary guidelines and higher diet quality [30].

For subsequent modeling, dietary variables were retained only for those with nonzero values among >50% participants at both baseline and 3 months. This resulted in 169 nutrients, 29 individual food subgroups, 20 collapsed food groups, and all measures of diet quality (HEI total and 13 HEI component scores) for analysis. Dietary variables included in the analyses are presented in Table S2.

#### 2.2.5. Plasma Targeted Metabolomics

Plasma samples obtained at clinic visits were assessed for a targeted panel of betaine, choline, carnitine, and trimethylamine oxide (TMAO) using liquid chromatography/mass spectroscopy (LC/MS) by the Mayo Clinic Metabolomics Core using previously published methods [31,32]. Plasma was spiked with D9-isotopes of internal standards prior to a cold methanol crash to remove proteins. The supernatant was dried and resuspended in running buffer for separation using a Grace Altima HP HILIC column (150 mm × 2.1 mm × 5 µm) on a Cohesive TX2 LC system (Franklin, MA). Samples were then introduced into a Sciex 6500 triple quadrupole mass spectrometer (Framingham, MA, USA) via electrospray ionization. Data acquisition was performed using selective ion monitoring mode, and concentrations of each analyte were determined using an 8-point standard curve for each respective analyte.

### 2.3. Statistical Analysis

Changes in indicators of health from baseline to 3 months were assessed via paired *t*-tests. Due to heteroscedasticity, laboratory measures were log-transformed prior to analysis. A two-step analysis process was used to assess associations between gut MB, DNAme, and dietary intakes (Figure 1). First, we tested the effect of MB taxa on DNAme cross-sectionally at baseline and 3 months. After removing samples with values ≥3 standard deviations from the mean for each feature, a linear model was fitted to test for the effect of individual MB taxa abundance on DNAme of all CpGs passing quality control while adjusting for age, sex, baseline BMI, predicted cell proportions, and two genetic principal components. This same analysis was completed for a subset of CpGs present in genes within metabolically relevant KEGG pathways as described above. Next, a linear model was fitted to test for the association between significant taxa from MB:DNAme analyses and dietary intake for each type of diet data separately (nutrients, food groups, diet quality) cross-sectionally at baseline and 3 months, as well as longitudinally over this time period. DNAme probes, MB taxa, and diet variables identified as significant in these analyses were further assessed for associations with plasma metabolites cross-sectionally at baseline and 3 months while adjusting for relevant covariates. All analyses were adjusted for multiple testing using the Benjamini–Hochberg false discovery rate (FDR) with a threshold for significance of 0.05 [33].

## 3. Results

### 3.1. Participant Demographic and Clinical Characteristics

Participants in this study (*n* = 47) were primarily non-Hispanic White females with a mean age of 40.9 ± 9.7 years (Table 1). At baseline, mean BMI was 33.5 ± 4.5 kg/m^2^, and individuals achieved significant weight loss on average, with the mean BMI decreasing by 2.1 ± 1.4 kg/m^2^ and waist circumference by nearly 9 cm (−8.5 ± 6.0 cm) at 3 months (both *p* < 0.001). Similar improvements were noted for plasma indicators of cardiometabolic health, including significant reductions in total cholesterol, triglycerides, glucose, and insulin from baseline to 3 months.

### 3.2. Integrated Microbiome and DNAme Analysis

**Figure 2:**
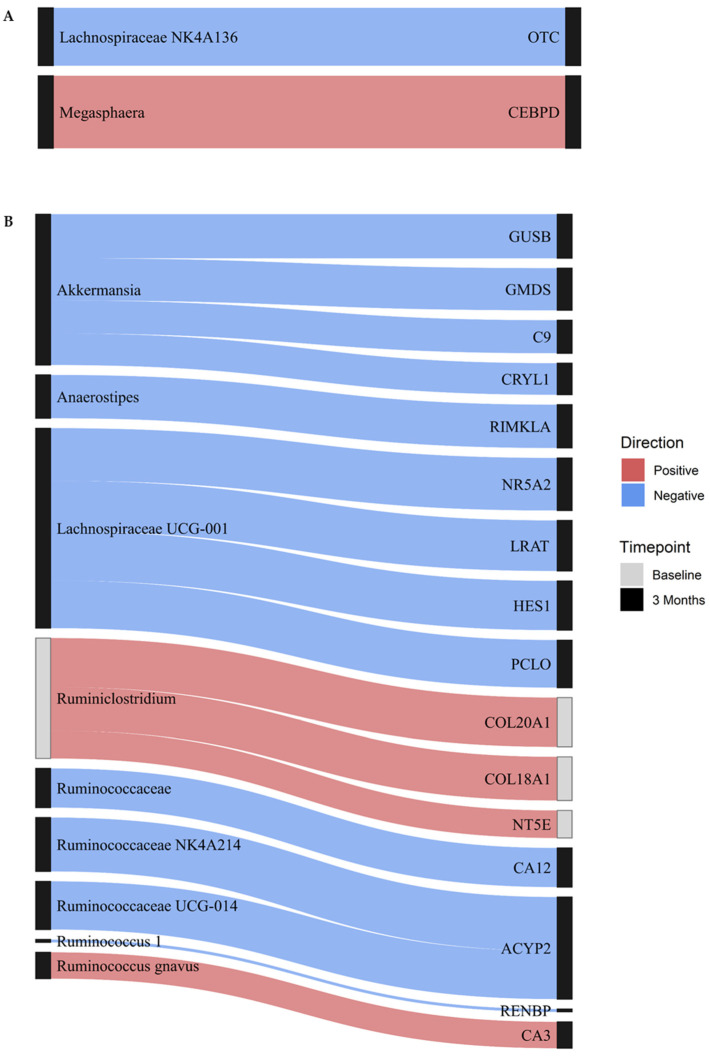
Cross-sectional associations between DNAme and gut microbiota. The Sankey plot displays associations between gut microbiota and DNAme of CpGs within labeled genes from (**A**) full and (**B**) reduced models at baseline (gray nodes) and 3 months (black nodes). Blue indicates an inverse association, whereas red indicates a positive association, while the thickness of each line indicates the strength of the association.

Integrated gut MB and DNAme analyses showed several significant cross-sectional associations (Table S3). In the full model containing all CpGs, there were no associations between gut MB and DNAme at baseline. At 3 months, there were two significant associations between the gut MB and DNAme (Figure 2A). These included a moderate inverse association between the abundance of *Lachnospiraceae NK4A136* and DNAme within an intron in Ornithine Transcarbamylase (*OTC*) and a strong positive association between the abundance of *Megasphaera* and DNAme in the promoter region of CCAAT/enhancer-binding protein delta (*CEBPD*).

In the reduced model using only CpGs in metabolically relevant KEGG pathways, three cross-sectional associations were noted at baseline (Figure 2B). Abundance of *Ruminiclostridium* was moderately positively associated with DNAme in the promoter region of 5′-Nucleotidase Ecto (*NT5E*), a gene involved in nicotinate and nicotinamide metabolism, as well as DNAme within intron regions of two collagen proteins involved in protein digestion and absorption, namely Collagen Type XVIII Alpha 1 Chain (*COL18A1*), and Collagen Type XX Alpha 1 Chain (*COL20A1*). Scatterplots displaying the underlying relationships for these three associations are presented in Figure S1. At 3 months, 14 unique MB:DNAme cross-sectional associations were observed (Figure 2B). Among these, abundance of *Akkermansia* was moderately inversely associated with DNAme in four genes: GDP-Mannose 4,6-Dehydratase (*GMDS*), complement 9 (*C9*), Crystallin Lambda 1 (*CRYL1*), and Glucuronidase Beta (*GUSB*). Similarly, *Lachnospiraceae UCG-001* was moderately inversely associated with DNAme in four genes: Nuclear Receptor Subfamily 5 Group A Member 2 (*NR5A2*), Hes Family BHLH Transcription Factor 1 (*HES1*), Piccolo Presynaptic Cytomatrix Protein (*PCLO*), and Lecithin Retinol Acyltransferase (*LRAT*). In the reduced model, *Ruminococcus gnavus* was the only MB taxon positively associated with DNAme at 3 months, demonstrating a significant association between abundance and DNAme in the gene Carbonic Anhydrase 3 (*CA3*). Scatterplots displaying the underlying relationships for these associations are presented in Figure S2.

### 3.3. Integrated Dietary Analysis

Dietary intakes were assessed among those with complete biospecimen collections and corresponding gut MB and DNAme data who also provided 7-day diet records at both baseline and 3 months (*n* = 40). Participants self-reported decreasing energy intakes by nearly 500 kcal/day (−479 ± 445 kcal/day) from baseline to 3 months (*p* < 0.001, Table 2). The percentage of calories from fat decreased by nearly 4%, while intakes from protein increased by a similar amount. Overall diet quality, as assessed by HEI total score, was also improved at 3 months (*p* = 0.022).

Integrated analysis between the DNAme-associated MB taxa and dietary intake data identified no associations between components of diet quality as measured by HEI. However, at 3 months, *Ruminococcaceae NK4A214* was associated with food-group level intakes of reduced-fat margarines. In change analyses, *Ruminococcus gnavus* was associated with dietary intakes of the nutrient, trans-octadecenoic acid, as well as total trans fatty acids, both of which significantly decreased from baseline to 3 months. 

### 3.4. Targeted Metabolomics Analysis

Plasma concentrations of betaine, choline, carnitine, and TMAO were not significantly associated with gut MB, DNAme, or dietary intakes at baseline or 3 months. 

## 4. Discussion

This study is one of few primary investigations of the association between the gut microbiome and epigenome in humans and the first to evaluate these relationships within the context of a behavioral weight loss intervention. At this 3-month early intervention time point, most individuals had lost a clinically significant amount of weight, showed improvements in several measures of cardiometabolic health, and had improved overall diet quality. Our results suggest several cross-sectional associations between the gut MB and DNAme in metabolically relevant pathways, providing evidence that gut MB composition correlates with epigenetic markers and, thus, could influence mechanisms underlying phenotypes observed before and during a weight loss intervention.

The only microbial taxon associated with the epigenome at baseline was *Ruminiclostridium*, a member of the Firmicutes (now renamed Bacillota) phylum and Ruminococcaceae family. The Ruminococcaceae family has been identified as one of primary differentiating characteristics among different gut MB enterotypes and was noted as a one of few family-level taxa that discriminate lean from obese phenotypes in a preclinical model of fecal transplantation from humans to mice [34,35,36]. *Ruminiclostridium* may be a beneficial component of the gut MB, as it is involved in secretion of short chain fatty acids (SCFAs) and plays a role in maintenance of intestinal epithelial cells [37]. In our analyses, baseline abundance of this taxon was positively associated with DNAme in three metabolically relevant genes. Expression of *COL18A1* has been associated with human adipocyte differentiation and susceptibility to obesity, and preclinical work demonstrates that knockout of *COL18A1* is associated with a reduction in adiposity in murine models [38,39]. A genome-wide meta-analysis linked the *COL20A1* gene to diabetic kidney disease [40]. The expression of *NT5E* has also been associated with the consumption of a high fat diet in preclinical models and, thus, may be associated with obesity [41]. The positive association between *Ruminiclostridium* abundance and DNAme in these genes indicates that this taxon may be associated with repression of these genes and may, therefore, play a beneficial role in regulation of adiposity among healthy adults with overweight or obesity. Future work should evaluate the association between measures of body fat and *Ruminiclostridium* abundance to test this hypothesis. 

Other members of the Ruminococcaceae family (*Ruminococcaceae uncultured*, *Ruminococcus 1*, *Ruminococcaceae NK4A214*, and *Ruminococcaceae UCG-014*) were inversely associated with DNAme in the promoter region of several genes involved in pyruvate, amino sugar and nucleotide sugar, and nitrogen metabolism at 3 months. Our previous analysis of multiomic predictors of weight loss in this cohort indicated that baseline *Ruminococcaceae UCG-014* was disadvantageous for weight loss, while *Ruminococcaceae NK4A214* and *Ruminococcus 1* were advantageous for weight loss and change in waist circumference [11,19], suggesting additional follow-up is needed to elucidate the mechanistic underpinnings of these relationships.

In the full model at 3 months, our analyses indicate a positive association between the abundance of *Megasphaera* and DNAme of the *CEBPD* gene, which is one of three CCAAT/enhancer-binding protein genes that have been implicated, along with peroxisome proliferator-activated receptor gamma (*PPARγ*) genes, as key regulators of adipogenesis [42,43]. Reduced expression of *CEBPD* has been associated with impaired adipocyte development. Furthermore, obesity has been associated with lower differentiation capacity of preadipocytes [44,45]. *Megasphaera*, a member of the Firmicutes (Bacillota) phylum, has been shown to be higher in individuals with obesity when compared to those of normal weight and has been positively correlated with body fat [46,47]. Thus, our data suggest that *Megasphaera* may be associated with epigenetic modification in pathways that could reduce the expression of major regulatory genes and impair appropriate development of adipocytes, leading to lower differentiation capacity and highlighting a potential mechanism by which the gut MB may influence the epigenome in obesity. 

At 3 months, *Akkermansia* was inversely associated with DNAme in four metabolically relevant genes. *Akkermansia* has demonstrated benefit in preventing high fat diet-induced obesity as well as in alleviating obesity-related insulin resistance and inflammation [48,49,50]. The most common *Akkermansia* species, *Akkermansia muciniphilia*, is a mucin-degrading and SCFA-producing bacterium that has been associated with lower body fat mass and improvements in multiple indicators of cardiometabolic health [51,52,53,54]. Though *Akkermansia* has been associated with beneficial changes in gene expression related to adipocyte differentiation and inversely correlated to gene variants associated with body mass index [53,55], to our knowledge, no previous studies have investigated the relationship between *Akkermansia* and DNAme in the genes identified in the present study. Thus, future work should characterize functional capacity of this taxon in relation to epigenetic markers and corresponding gene expression, particularly in the context of behavioral weight loss interventions for the management of overweight and obesity. 

Integrated analyses evaluating the relationship between the gut MB and epigenome demonstrated inverse cross-sectional associations between the abundance of *Lachnospiraceae* and DNAme in several genes at both baseline and 3 months. This family is within the Firmicutes (Bacillota) phylum and includes a heterogenous group with diverse functions, with some studies suggesting a beneficial effect through mechanisms, such as enhanced SCFA production, and others finding associations with metabolic disease [10,56,57]. Interestingly, research indicates that a greater abundance of some *Lachnospiraceae* species is associated with obesity and altered lipid and glucose metabolism, while others, such as *Lachnospiraceae NK4A136*, have been described as protective against obesity, with potential anti-inflammatory effects and previously reported associations with greater adherence to healthy dietary patterns, such as a Mediterranean diet [56,58,59]. 

At baseline, a greater abundance of *Lachnospiraceae NK4A136* was correlated with hypo-methylation in the *OTC* gene, which plays a role in mitochondrial metabolism, amino acid homeostasis in the liver and small intestine, and has been associated with insulin response [60]. Decreased *OTC* expression has also been linked to reduced enterocyte mass and function as well as poorer liver function, though it is unclear if these are causes or consequences of underlying disease. At 3 months, *Lachnospiraceae UCG-001* abundance was inversely associated with DNAme in the promoter region of four metabolically relevant genes, including those involved in insulin secretion. While some work indicates that a greater abundance of *Lachnospiraceae UCG-001* may be associated with obesity, other preclinical and human studies suggest this species may be predictive of weight loss among those with overweight/obesity [61,62]. In our previous analyses of associations between gut MB and cardiometabolic health indicators in this cohort, we observed that greater abundance of an unclassified member of *Lachnospiraceae* was associated with less decrease in waist circumference from baseline to 3 months [11,19]. However, as detailed above, findings from our current analyses suggest different *Lachnospiraceae* species may be beneficial in modulating epigenetic changes, suggesting further investigation at the sequence variant level is warranted. These inconsistencies may be a result of using 16S data versus shotgun metagenomics, or they could be due to substantial inter-individual variation in the structure and function of the gut MB, highlighting the individualized role some taxa may play in metabolic regulation. 

While dietary patterns have been suggested to be more closely associated with gut MB composition than individual nutrients [63,64], no associations with metrics of overall diet quality, nor expected dietary components, such as fiber, or food groups, such as grains, vegetables, or dairy, were noted when gut MB was evaluated alongside dietary intake data in our cohort. This may be because the gut MB is highly personalized in its response to dietary intake, with few diet–MB interactions conserved across people [65,66]. In addition, food composition databases underlying dietary analysis software capture only a fraction of the components within foods that may affect the gut MB [67]. Previous analyses have also demonstrated that habitual or long-term dietary intake is more closely associated with gut MB composition than intake reported from shortly before MB sampling [36], and, thus, use of food frequency questionnaires or weighted daily intake data may be useful for identifying relationships between gut MB and diet. Of note, however, is that an increased abundance of *Ruminococcus gnavus* was associated with decreases in intakes of trans fatty acids from baseline to 3 months. Previous studies have implicated *Ruminococcus gnavus*, which is a mucin-degrading group, in inflammatory bowel disease and in altered lipid metabolism related to non-alcoholic fatty liver disease (NAFLD) and obesity [46,68,69]. Relatedly, our analysis of multiomic predictors of change in weight and cardiometabolic health in this cohort indicate that this taxon is disadvantageous for reductions in triglycerides [19]. Furthermore, though not observed in this study, this taxon has been inversely associated with long-term diet quality, as assessed by HEI-2015 and positively associated with consumption of a pro-inflammatory diet in a large, multiethnic cohort [70,71], suggesting abundance may be higher among those with suboptimal dietary intake.

The strengths of this study include a rigorously designed weight loss trial with intensive behavioral support and careful monitoring of dietary intake by RDNs using multi-day diet records and comprehensive diet analysis software. In addition, blood and fecal sample collection aligned with other assessments and were analyzed for DNAme and gut MB, respectively, using validated and widely employed methods. Despite these strengths, this study is not without limitations. The participants included in this analysis represent two of five recruitment cohorts for the parent trial, and, thus, the sample size is relatively small and not representative of the general population of those with overweight and obesity in terms of race, ethnicity, socioeconomics, and other factors. Use of short-read 16S rRNA amplicon sequencing for the evaluation of gut MB composition limited our ability to evaluate functional capacity, and assessment of DNAme in whole blood did not allow for comparisons with metabolically active tissues. Furthermore, inclusion of transcriptomics, proteomics, or more thorough characterization of metabolomics in future studies would help to further elucidate relationships with the gut MB. Observations may have been related to unmeasured factors, such as physical activity or change in fat mass at 3 months. Lastly, while current best practice, self-reported dietary intake is prone to innate bias [72]; thus, underreporting, particularly for energy intake, may be likely within this population. 

## 5. Conclusions

Healthy adults with overweight or obesity enrolled in a behavioral weight loss program demonstrate changes in body weight, cardiometabolic health, and dietary intakes after 3 months of intervention. At baseline and this early intervention time point, there are several associations between the gut MB and DNAme in genes known to be involved in KEGG pathways related to obesity and metabolism. Thus, the identified relationships may provide initial insights into potential pathways through which changes in the gut MB may affect metabolism before and during weight loss. Though relationships were not associated with overall dietary patterns or a subset of gut MB-derived metabolites, such as TMAO, future work may consider modeling other dietary features or integration of untargeted metabolomics to further explore the mechanisms by which the gut MB and epigenome interact to influence weight status. Furthermore, this study provides a foundation for the co-evaluation of multiomics in lifestyle interventions to discern associations between modifiable factors that may be useful for identifying baseline predictors of response, drivers of interindividual metabolic variability, or future therapeutic targets.

## Figures and Tables

**Table 1:** Participant demographic characteristics at baseline and 3 months (*n* = 47).

Characteristic		Baseline Mean ± SD or % (*n*)	3 Months Mean ± SD or % (*n*)	Change (*p* Value)
Age, years		40.9 ± 9.7	-	-
Sex	Male	23 (11)	-	-
	Female	77 (36)	-	-
Race	White	89 (42)	-	-
	Black	6 (3)	-	-
	Multiracial	4 (2)	-	-
Ethnicity	Hispanic	9 (19)	-	-
	Non-Hispanic	81 (38)	-	-
Income	<$25,000 USD	11 (5)	-	-
	$25,000–$45,000 USD	4 (2)	-	-
	$45,001–$70,000 USD	23 (11)	-	-
	$70,001–$110,000 USD	25 (12)	-	-
	>$110,000 USD	36 (17)	-	-
Education	Some college	11 (5)	-	-
	Four-year degree	45 (21)	-	-
	Master’s degree	34 (16)	-	-
	Doctorate degree	11 (5)	-	-
Weight, kg		96.1 ± 16.1	90.2 ± 15.3	−6.0 ± 3.9
				(<0.001)
Body mass index, kg/m^2^		33.5 ± 4.5	31.5 ± 4.3	−2.1 ± 1.4
				(<0.001)
Waist circumference, cm		109.4 ± 10.3	100.9 ± 10.5	−8.5 ± 6.0
				(<0.001)
Systolic blood pressure, mmHg		117 ± 14	114 ± 12	−3 ± 12
				(0.104)
Diastolic blood pressure, mmHg		74 ± 8	76 ± 9	2 ± 12
				(0.173)
Total cholesterol, mg/dL ^a^		180 ± 34	165 ± 30	−15 ± 26
				(<0.001)
High-density lipoprotein (HDL) cholesterol, mg/dL ^a^		48 ± 12	47 ± 12	−1 ± 6
				(0.288)
Triglycerides, mg/dL ^a^		136 ± 79	107 ± 56	−29 ± 61
				(0.002)
Glucose, mg/dL ^a^		93 ± 11	88 ± 8	−5 ± 11
				(0.002)
Insulin, uIU/mL ^a^		12 ± 8	7 ± 5	−4 ± 6
				(<0.001)

^a^ log-transformed prior to analysis, *n* = 45 due to missing data.

**Table 2:** Participant dietary intakes at baseline and 3 months (*n* = 40).

Characteristic	Baseline Mean ± SD	3 Months Mean ± SD	Change (*p* Value)
Energy (kcal/day)	1764 ± 338	1284 ± 380	−479 ± 445 (<0.001)
Carbohydrate (% kcal)	42 ± 8	42 ± 7	0 ± 6 (0.797)
Fat (% kcal)	39 ± 7	35 ± 5	−4 ± 6 (<0.001)
Protein (% kcal)	17 ± 3	21 ± 4	4 ± 5 (<0.001)
Fiber (g/day)	16 ± 5	14 ± 6	−2 ± 6 (0.015)
Diet quality (total HEI score)	57 ± 12	62 ± 12	4 ± 12 (0.022)

## Data Availability

The gut microbiome data used in this study are available from the European Bioinformatics Institute (EBI), accession No. PRJEB64902 (ERP150076). Methylation data have been deposited in the Gene Expression Omnibus and are publicly available through accession GSE240184.

## Supplementary Materials

The following supporting information can be downloaded at: https://www.mdpi.com/article/10.3390/nu15163588/s1, Table S1: 130 metabolically relevant KEGG pathways for inclusion in sensitivity analyses; Table S2: Dietary variables from the Nutrition Data System for Research employed for integrated diet and microbiome analyses; Table S3: Full and reduced model results for integrated DNAme and microbiome analyses; Figure S1: Scatterplots of baseline cross-sectional associations between DNAme in genes within metabolically relevant KEGG pathways and gut microbial taxa abundance; Figure S2: Scatterplots of 3-month cross-sectional associations between DNAme in genes within metabolically relevant KEGG pathways and gut microbial taxa abundance.
