# Supplementary material for: The Microbiome, Epigenome, and Diet in Adults with Obesity during Behavioral Weight Loss
Source: Nutrients. 2023 Aug 16;15(16):3588. doi: 10.3390/nu15163588 (PMC10458964; doi:10.3390/nu15163588)

Figure S1. Scatterplots of baseline cross-sectional associations between DNAme in genes within metabolically-relevant KEGG pathways and gut microbial taxa abundance

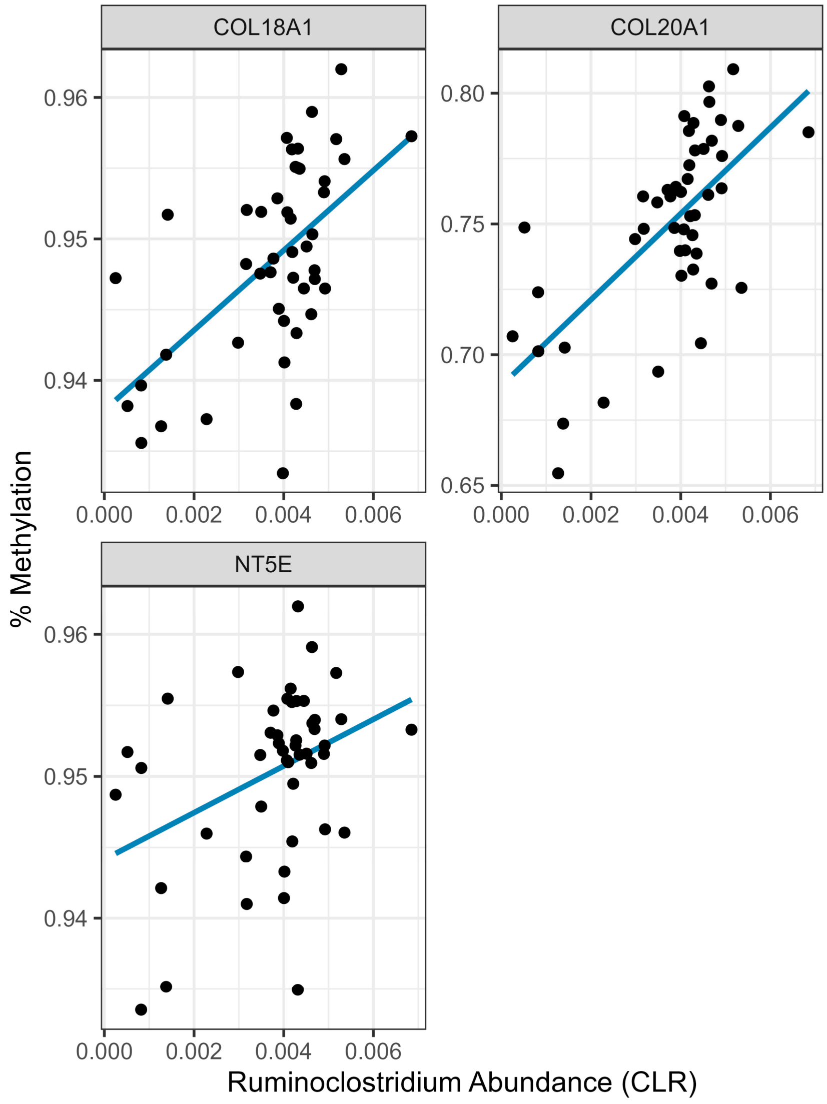

Figure S2. Scatterplots of 3-month cross-sectional associations between DNAme in genes within metabolically-relevant KEGG pathways and gut microbial taxa abundance

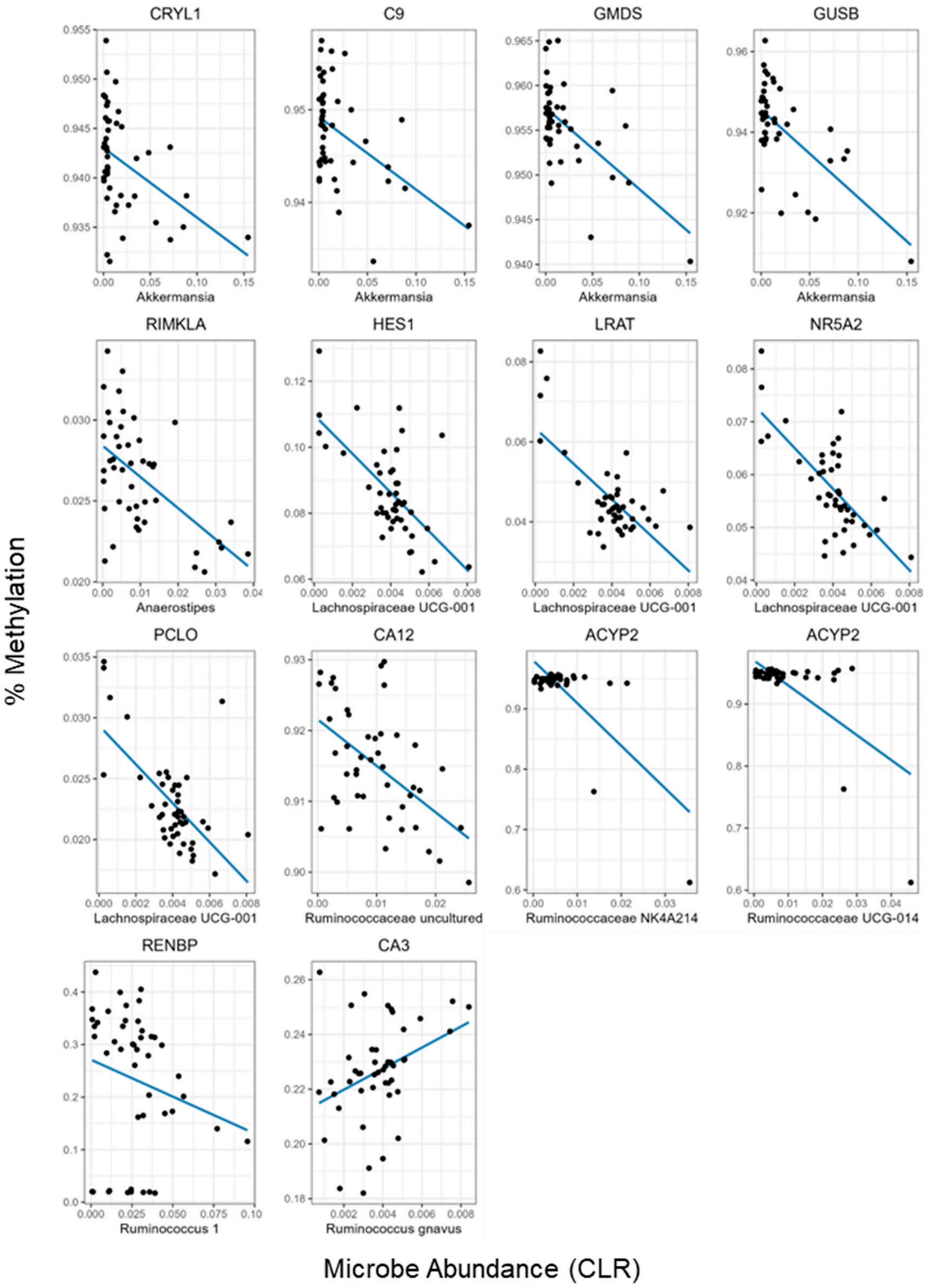

Supplement: Supplementary file 1 [file nutrients-15-03588-s001.zip › nutrients-2483258-supplementary/HillKonigsberg_GutMBDNAmeDiet_Nutrients_SupplementaryFigures 230616.pdf]
